# Foliar δ^13^C Showed No Altitudinal Trend in an Arid Region and Atmospheric Pressure Exerted a Negative Effect on Plant δ^13^C

**DOI:** 10.3389/fpls.2017.01070

**Published:** 2017-07-04

**Authors:** Zixun Chen, Guoan Wang, Yufu Jia

**Affiliations:** College of Resources and Environmental Sciences, China Agricultural UniversityBeijing, China

**Keywords:** plant δ^13^C, altitude, atmospheric pressure, temperature, arid mountainous terrain

## Abstract

Previous studies have suggested foliar δ^13^C generally increases with altitude. However, some observations reported no changes or even decreased trends in foliar δ^13^C. We noted that all the studies in which δ^13^C increased with elevation were conducted in the human regions, whereas those investigations in which δ^13^C did not vary or decreased were conducted in areas with water stress. Thus, we proposed that the pattern of increasing δ^13^C with elevation is not a general one, and that δ^13^C may remain unchanged or decrease in plants grown in arid environments. To test the hypothesis, we sampled plants along altitude gradients on the shady and sunny slopes of Mount Tianshan characterized by arid and semiarid climates. The measurements of foliar δ^13^C showed no altitudinal trends for the plants grown on either of the slopes. Therefore, this study supported our hypothesis. In addition, the present study addressed the effect of atmospheric pressure on plant δ^13^C by accounting for the effects of temperature and precipitation on δ^13^C. This study found that the residual foliar δ^13^C increased with increasing altitude, suggesting that atmospheric pressure played a negative role in foliar δ^13^C.

## Introduction

The ^13^C isotope of atmospheric CO_2_ is discriminated against during photosynthetic carbon fixation. The carbon isotope ratio (δ^13^C) in plants depends on the ratio of intercellular to ambient CO_2_ concentration (*c*_i_/*c*_a_), which reflects the balance between the rate of inward CO_2_ diffusion, mediated by stomatal conductance (*g*), and the rate of CO_2_ assimilation (A) (Farquhar and Richards, 1984; Cernusak et al., 2013), and has been suggested as a sensitive long-term indicator of physiological changes. Considerable effort has been invested in observing and understanding the variation in δ^13^C along environmental gradients and the effects of environmental factors on δ^13^C (e.g., Diefendorf et al., 2010; Kohn, 2010; Wang et al., 2013; Broeckx et al., 2014; Xu et al., 2015; Yang et al., 2015; Čada et al., 2016).

A pattern of increasing δ^13^C with increasing altitude has consistently been reported previously (Körner et al., 1988, 1991; Sparks and Ehleringer, 1997; Li et al., 2009). However, exceptions to the general pattern of increasing δ^13^C with altitude have been observed. Friend et al. (1989), Lajtha and Getz (1993), and Van de Water et al. (2002) found that δ^13^C decreased with altitude; Wang et al. (2010) reported no altitudinal trends in plants δ^13^C. We noted that most of the studies in which a pattern of increasing δ^13^C with elevation were concentrated in regions where water was not a factor limiting plant growth, whereas those studies with the opposite pattern and/or no altitudinal trend were conducted in arid or semiarid regions. Thus, we argued that the pattern of increasing δ^13^C with elevation is not a general one, and that altitudinal variation in plants δ^13^C depends on soil moisture. We proposed that altitudinal variation in δ^13^C might be negative or unchanged for plants grown in arid or semiarid regions.

The altitudinal trend of plant δ^13^C is possibly attributable to variations in various environmental factors, such as temperature, precipitation, atmospheric pressure, and solar radiation. Temperature and/or precipitation have consistently been considered the main contributors. However, the contribution of atmospheric pressure is inconclusive. Körner et al. (1991), Kelly and Woodward (1995), and Zhu et al. (2010) suggested that decreasing atmospheric pressure with altitude played an important role in the altitudinal variations in plants δ^13^C. Sparks and Ehleringer (1997) argued that atmospheric pressure was a secondary factor even if it has an effect. Previous studies were unable to effectively segregate the influence of atmospheric pressure from the effects of precipitation and temperature. Thus, accounting for the effects of temperature and precipitation on plant δ^13^C may yield a reliable relationship between atmospheric pressure and δ^13^C.

In this study, we investigated the variation in foliar δ^13^C with altitude on the sunny and shady slopes of Mount Tianshan, a typical arid mountainous terrain, in Xinjiang Uygur Autonomous Region, China. The first objective was to test our hypothesis that δ^13^C decreases or remains unchanged for plants grown in arid and semi-arid regions. The second objective was to determine the effect of atmospheric pressure on plant δ^13^C through accounting for the effects of temperature and precipitation.

## Materials and Methods

### Study Site

**FIGURE 1:**
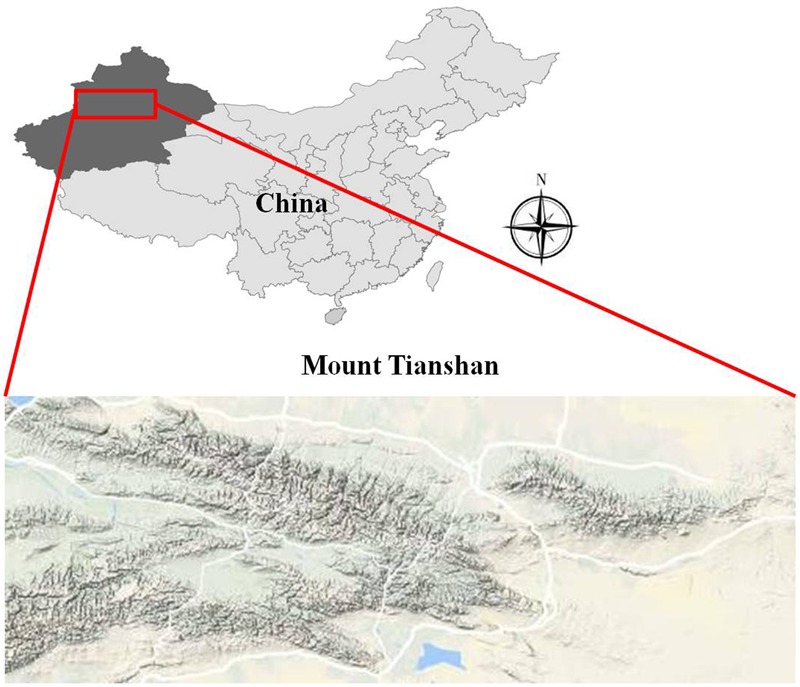
The location of the study area.

Mount Tianshan, the maximal mountains in arid region all over the world, is located inside Eurasia and stretches over four countries including China, Kazakhstan, Kyrgyzstan, and Uzbekistan. In China, Mount Mountain is located in the middle of the Xinjiang Uygur Autonomous Region. It covers about 570,000 km^2^ and is 1,700 km long in an east-west direction and accounts for a third of the whole area of Xinjiang (**Figure 1**).

Our study site was located on eastern Mount Tianshan (42.43–43.53°N, 86.23–87.32°E) (**Figure 1**), along an elevation transect on the shady and sunny slopes. Mount Tianshan is characterized by a mountainous climate; vertical variations in temperature and precipitation are very pronounced, temperature decreases and precipitation increases with altitude on both slopes. The shady northern slope of Mount Mountain is controlled by air masses from the Arctic and Atlantic Ocean, so it is colder and moister than the sunny southern slope, which is warmer and more arid because the mountain hinders the humid air. The average annual mean temperature (AMT) is -1.85 and 1.03°C, and the average annual mean precipitation (AMP) is 402 and 246 mm on the shady and sunny slopes, respectively. Along our elevation transects, there were four meteorological observatories; two were on the shady slope and the other two were on the sunny slope (**Table 1**). The vertical distributions of vegetation and soil spectrums were also very pronounced on the two slopes. The detailed information is shown in **Table 2**.

**Table 1:** Data of climate from the meteorological observatories in the research area.

Meteorological observatories	Locations	AMT/°C	AMP/mm	Atmospheric pressure/hPa	Alt./m
WLMQ	Northern slope	6.9	269.4	912.1	918.7
MOS	Northern slope	-5.2	453.4	653.5	3539.0
BLT	Southern slope	6.6	208.4	834.9	1738.3
YQ	Southern slope	8.4	73.3	903.6	1055.8

*WLMQ, Wulumuqi Meteorological Observatory; MOS, Mountain Observation Station of the Tianshan Glaciological Station, Chinese Academy of Sciences; BLT, Baluntai Meteorological Observatory; YQ, Yanqi Meteorological Observatory; AMT, annual mean temperature; AMP, annual mean precipitation.*

**Table 2:** The vertical distributions of vegetation and soil on Mount Tianshan.

Northern slope			Southern Slope		
Altitude (m)	Vegetation	Soil	Altitude (m)	Vegetation	Soil
800–1100	Upland desert	Brown calcic soil	1300–1800	Upland desert	Sierozem
1100–2500	Upland steppe	Chestnut soil	1800–2600	Arid upland steppe	Chestnut soil
1800–2700	Frigid coniferous forest	Mountain gray cinnamon forest soil	2600–2800	Subalpine steppe	Chestnut soil
2500–3300	Subalpine meadow	Subalpine meadow soil	2800–3800	Alpine meadow and cushion plants	Alpine meadow soil
3300–3700	Alpine meadow	Alpine meadow soil	3800–4000	Alpine desert zone	Chilly desert soil
3700–3900	Alpine sparse vegetation and a desert zone	Chilly desert soil	Above 4000	Alpine ice-and-snow zone	Chilly desert soil
Above 3900	Alpine ice-and-snow zone	Chilly desert soil			

### Samplings of Plants and Soil

An altitudinal transect of 1,564–3,800 m above sea level (a.s.l.) was set on the shady slope, and 1,300–3,780 m a.s.l. on the sunny slope. Plants and soil were collected along the two transects at intervals of about 100 m. The sampling was restricted to open sites that were far from the major roads and human habitats. The sampling was conducted in July 2014. A total of 5–7 individual plants of each species were collected and the same number of leaves was sampled from each individual plant. For shrubs and herbs, the uppermost leaves of each individual plant were collected; for tree species, eight leaves were collected from each individual, two leaves were collected at each of the four cardinal directions from the positions of full irradiance, about 8–10 m above the ground. The leaves from the same species of each site were combined into one sample. A total of 105 plant samples were collected on the shady slope, belonging to 21 families and 37 genera, most of which were herbs (only 15 were woody plants); the main species were *Kobresia myosuroides, Carex enervis, Poa annua*, and *Thalictrum aquilegifolium*. One hundred and twelve samples were collected on the sunny slope, belonging to 16 families and 39 genera (only 18 were woody plants); the main plants were *Stipa grandis, Stipa capillata, Achnatherum splendens*; *Artemisia frigida, Nitraria tangutorum, Caragana sinica*, and *Suaeda glauca*. No C_4_ plants occurred on the shady slope, and only four C_4_ plant samples were collected on the sunny slope; thus, the altitudinal variation in C_4_ plant δ^13^C was not considered in this study. At each sampling site, we collected three surface soil samples (0–5 cm depth) using a ring after removing the litter layer within a radius of 20 m. These soil samples were used to determine soil density and water moisture.

### Measurements of Foliar δ^13^C

Plant samples were air-dried in the field and then in the laboratory. Leaves were then ground into a fine powder using a steel ball mixer mill MM200 (Retsch GmbH, Haan, Germany). Approximately 0.35 mg leaf powder was wrapped up in a tin capsule, and was sent into an oxidation furnace of an elemental analyzer (EA) (FlashEA 1112; CE Instruments, Wigan, United Kingdom) through an autosampler. This EA was coupled with a Delta^Plus^ XP mass spectrometer (Thermo Scientific, Bremen, Germany). The leaf powder was combusted in the oxidation furnace at 1,020°C with chromium oxide as the oxidizing agent and silvered cobaltous oxide as the catalyst. The gaseous products of this combustion, including carbon dioxide, nitric oxide, water vapor, etc., were pulled into a reduction furnace of the EA by He, a carrier gas. In the reduction furnace, nitric oxide reacted with copper to form N_2_; carbon dioxide and water remained unchanged. N_2_, carbon dioxide, and water vapor then passed through a water trap of the EA where water vapor was absorbed by magnesium perchlorate. The remaining N_2_ and carbon dioxide flowed into a chromatographic column of the EA where they were separated. Finally, only CO_2_ was allowed to enter the Delta^Plus^ XP mass spectrometer where the carbon isotope of CO_2_ was determined. The carbon isotopic ratios were reported in the delta notation relative to the V-PDB standard. For this measurement, we obtained standard deviations of <0.15‰ for δ^13^C among replicate measurements of the same sample.

### Measurements of Soil Water

**FIGURE 2:**
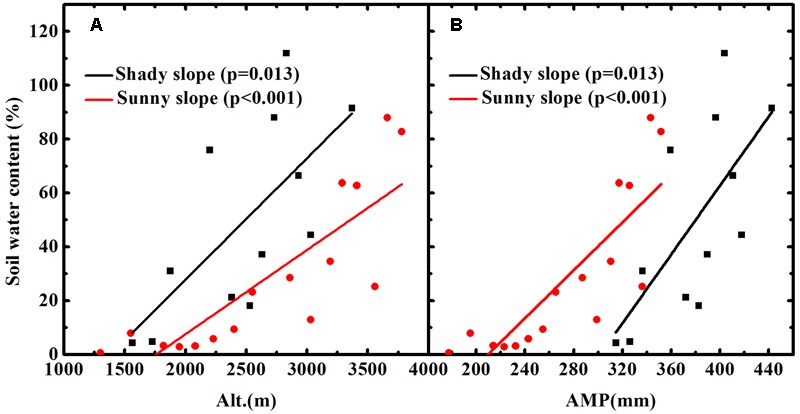
Soil water contents vary with altitude **(A)** and annual mean precipitation (AMP) **(B)** in Mount Tianshan.

The soil water content was determined after oven drying at 105 ± 2°C to a constant weight. The soil water content of each sample was the difference between its wet and dry weight divided by its dry weight. An error occurring during the transport from the field to laboratory resulted in an incomplete set of soil moisture data for the shady slope. However, we obtained a complete set of soil moisture data for the sunny slope. Soil moisture increased with altitude (**Figure 2A**) and was positively related to AMP for the soil on the sunny slope (**Figure 2B**). The incomplete soil moisture data of the shady slope also showed the same pattern as the soil of the sunny slope (**Figures 2A,B**). Although the higher elevations did not have soil water content data on the shady slope, we firmly believe the pattern of increasing soil moisture with altitude is applicable for the all the soil of the shady slope because we observed that the soil at higher elevations was clearly wetter than the soil at lower elevation during field sampling.

### Accounting for the Effects of Temperature and Precipitation on Plant δ^13^C

As presented in the introduction, to better understand the correlation between atmospheric pressure and plant δ^13^C, we have to eliminate the effects of temperature and soil water availability on plant δ^13^C. In this study, we used AMP to replace soil water availability to correct the influence of soil moisture based on three reasons. First is that the coefficient of plant δ^13^C vs. soil moisture has not previously been established to our knowledge; in contrast, the coefficient of plant δ^13^C vs. AMP has been reported in many previous studies. Second, as shown in **Figure 2B**, soil water availability generally displays a positive correlation with AMP. Third, although soil moisture is a more direct factor influencing plant δ^13^C compared to AMP, AMP could more fully reflect long-term soil moisture status relative to the soil moisture data derived from only one or several measurements. We assumed that the effects of temperature and precipitation are linearly additively and there are no cross effect for more than one variable. Regarding the effect of precipitation on plant δ^13^C, previous studies mostly suggested that it was negative (Stewart et al., 1995; Wang et al., 2008; Gebrekirstos et al., 2009; Diefendorf et al., 2010; Kohn, 2010; Sayak et al., 2015), although the coefficient of precipitation vs. δ^13^C obtained in previous investigations was not exactly the same. The correction of precipitation effect was conducted by using a rate of -0.4‰/100 mm reported by Wang et al. (2008). Both the present study and that of Wang et al. (2008) were conducted in northwest China; although the plant species in the two studies were not identical, the majority of plant species occurring in the study area of this investigation could be found in the place of Wang et al. (2008), because the climate, especially precipitation is very similar between the two areas. For example, about 99% of the plant samples in Wang et al. (2008) were collected from areas with an AMP from 180 to 630 mm, whereas in the present study, the AMP varied from 177 to 564 mm. Therefore, we believe that the coefficient of δ^13^C vs. AMP obtained in Wang et al. (2008) is suitable for the correction of precipitation in this study. In addition, even if there is a slight variation in the coefficient of δ^13^C vs. AMP, this will not make a significant difference in our final results. However, it is difficult to determine whether accounting for the temperature effect is necessary, and if so, how much correction should be made, because no conclusive statement of the temperature effect has been reached as yet. All three patterns, positive, negative, and no influence, have been reported (e.g., Heaton, 1999; Schleser et al., 1999; McCarroll and Loader, 2004; Treydte et al., 2007; Gebrekirstos et al., 2009; Wang et al., 2013; Trahan and Schubert, 2016). Thus, all three patterns should be taken into account when making a temperature-correction. However, we did not consider the negative and no effects based on two reasons. First, most of the previous studies showed a positive correlation between temperature and plant δ^13^C; second, no suitable δ^13^C-temperature coefficient could be used because negative coefficients were derived mostly from tree-ring δ^13^C series, in which only one or a few trees were used. Under the situation with the correction of positive temperature effect, we used the coefficient of δ^13^C-AMT of Wang et al. (2013) (0.104‰/°C). Wang et al. (2013) was conducted along a temperature gradient with 400 mm annual precipitation across China, so the effect of precipitation on δ^13^C was minimized. Furthermore, the coefficient of δ^13^C-AMT reflected the mean variation in plant δ^13^C with temperature covering 283 plant species including herbs, shrubs, and woods, thus having a high representation of all types. In addition, the areas along the 400 mm isopleth of annual precipitation across China are characterized by a semiarid climate analogous to that of Mount Tianshan. The values of AMT and AMP at each sampling site were obtained through linear interpolation based on climatic data from two meteorological observatories on the shady slope and two meteorological observatories on the sunny slope.

### Statistical Analysis

Statistical analyses were conducted using SPSS software (SPSS for Windows, Version 20.0, Chicago, IL, United States). One-way analysis variance (ANOVA) was used to compare δ^13^C in all plants and herbs between shady and sunny slopes. The correlation analyses of altitude vs. the original δ^13^C and altitude vs. the residual δ^13^C after accounting for the effects of temperature and precipitation were performed to determine the effect of altitude and atmospheric pressure on plant δ^13^C.

## Results

**FIGURE 3:**
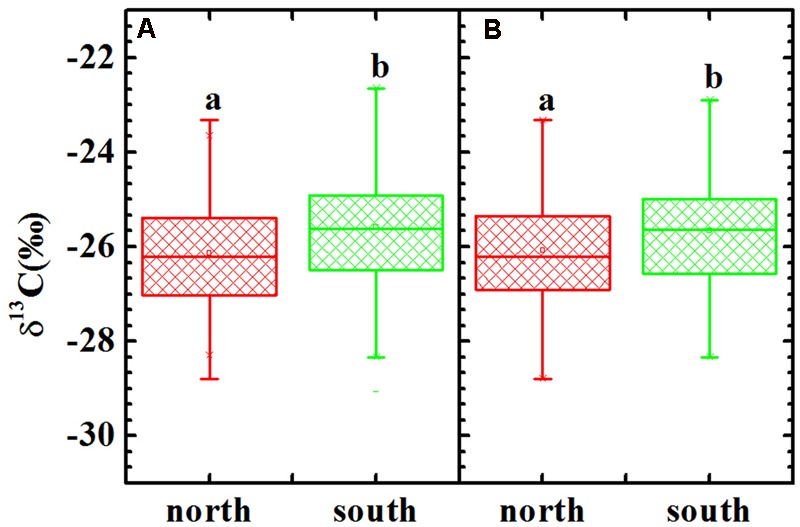
δ^13^C values of all C_3_ plants **(A)** and herbs **(B)** in the shady and sunny slopes of Mount Tianshan. Different letters indicate a significant difference (*p* < 0.05).

The δ^13^C values ranged from -23.33 to -28.80‰ with a mean value of -26.13‰ (*n* = 105) for the C_3_ plants on the shady slope, whereas it ranged from -22.12 to -29.06‰ with a mean value of -25.57‰ (*n* = 108) for the C_3_ plants on the sunny slope (**Figure 3A**). A one-way ANOVA suggested a significant difference between the two slopes for all C_3_ plants (*p* = 0.001). Considering the possible effect of life-forms on plant δ^13^C, the comparison between the two slopes for herb δ^13^C was also performed, and a one-way ANOVA showed a similar pattern to that observed for all C_3_ plants (**Figure 3B**, *p* = 0.008). A comparison for woody plants was not conducted owing to limited samples.

**FIGURE 4:**
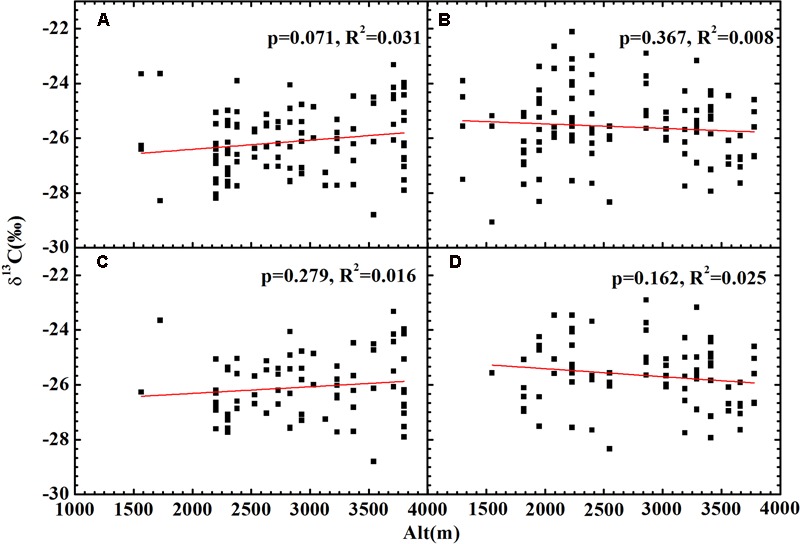
The relationships between plant δ^13^C and altitude. **(A)** All plants on the shady slope; **(B)** all plants on the sunny slope; **(C)** herbs on the shady slope; **(D)** herbs on the sunny slope. Correlation analyses showed no significant relationships for **(A–D)**.

**FIGURE 5:**
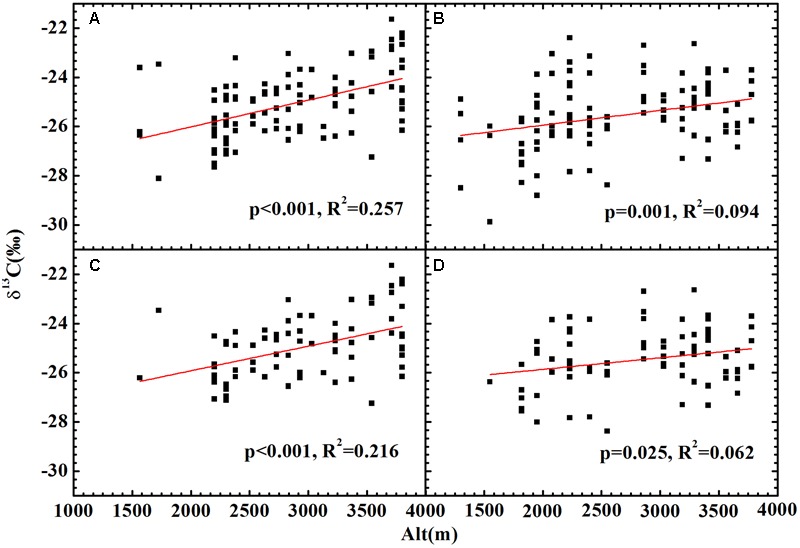
The relationships between altitude and the plants δ^13^C after being accounted for the effects of temperature and precipitation. **(A)** All plants on the shady slope (δ^13^C = 1.09 × 10^-3^Alt-28.19, *R*^2^ = 0.257, *p* < 0.001); **(B)** all plants on the sunny slope (δ^13^C = 0.60 × 10^-3^Alt-27.14, *R*^2^ = 0.094, *p* = 0.001); **(C)** herbs on the shady slope (δ^13^C = 0.99 × 10^-3^Alt-27.91, *R*^2^= 0.216, *p* < 0.001); **(D)** herbs on the sunny slope (δ^13^C = 0.45 × 10^-3^Alt-26.80, *R*^2^ = 0.062, *p* = 0.025).

No significant relationship between altitude and δ^13^C was found for all plants (**Figures 4A,B**), herbs (**Figures 4C,D**) or woody plants (Supplementary Figure 1) growing on both slopes. After accounting for the combined effects of temperature and precipitation, the residual δ^13^C exhibited a strong positive correlation with altitude for all plants (**Figures 5A,B**) and herbs (**Figures 5C,D**). The residual δ^13^C increased with altitude with a coefficient of 1.09‰/km for all plants on the northern slope, 0.60‰/km for all plants on the southern slope, 0.99‰/km for the herbs on northern slope, and 0.47‰/km for the herbs on southern slope. It is well known that there is always an extremely significant relationship between air pressure and altitude (Supplementary Figure 2), although the change of air pressure is a function of temperature. Thus, the above results suggest an increase in δ^13^C with decreasing atmospheric pressure.

## Discussion

One-way ANOVA analyses showed that the plants grown on the sunny slope enriched more ^13^C than that on the shady slope; the drier and warmer climate on the sunny slope relative to the shady slope accounted for this result. δ^13^C did not change with altitude for all plants and herbs grown on both slopes, indicating that the pattern of no altitudinal trend of plant δ^13^C in arid and semi-arid regions is not dependent on microclimate and topography; furthermore, it may also be irrespective of the plant life form. The results confirmed our hypothesis. This observation, combined with previous investigations (Friend et al., 1989; Lajtha and Getz, 1993; Van de Water et al., 2002; Wang et al., 2010), strongly suggested that increasing δ^13^C with increasing elevation is only true for plants grown in humid regions.

The altitudinal variation in plant δ^13^C has been suggested to be correlated with changes in temperature, precipitation, atmospheric pressure, and solar irradiation with elevation. In humid regions, water availability is high and possibly plays a secondary role in carbon isotope discrimination (Körner et al., 1988, 1991; Sparks and Ehleringer, 1997). However, for plants in arid or semiarid habitats, soil moisture is the key growth-limiting factor. Xu et al. (2015) showed that the key growth-limiting factor is also crucial for ^13^C discrimination; therefore, water availability is possibly the most significant factor determining plant δ^13^C in regions with water stress. Thus, it could explain differences in the altitudinal pattern of plant δ^13^C between regions with water deficits and humid areas. Although there is a difference in precipitation between the two slopes of Mount Tianshan, soil moisture is a key growth-limiting factor for the plants on the two slopes, so the same pattern of altitudinal variations in plant δ^13^C was found on the sunny and shady slopes.

Except in extreme wet environments, the effect of water availability on δ^13^C is generally negative (e.g., Ehleringer and Cooper, 1988; Stewart et al., 1995; Wang et al., 2008; Diefendorf et al., 2010; Kohn, 2010). Precipitation increases with altitude on the northern and southern slopes in Mount Tianshan (**Table 1**); therefore, the altitudinal trend of precipitation should result in more negative δ^13^C with increasing elevation on Mount Tianshan. With more water availability, stomatal conductance increases, which leads to CO_2_ entering the internal leaf, and consequently the *c*_i_/*c*_a_ ratio increases and thus δ^13^C decreases.

The effect of temperature on plant δ^13^C has been assessed intensively. Studies of C_3_ plants suggested mostly positive correlations between δ^13^C and temperature (e.g., Stuiver and Braziunas, 1987; Treydte et al., 2007; Wang et al., 2013; Soolanayakanahally et al., 2015; Trahan and Schubert, 2016), although some negative (Morecroft and Woodward, 1990, 1996; Sheu and Chiu, 1995; Edwards et al., 2000) and no significant (Troughton and Card, 1975; Gebrekirstos et al., 2009; Diefendorf et al., 2010) correlations have been reported. The altitudinal trend of plant δ^13^C is attributable mainly to the negative effect of temperature on δ^13^C (Körner et al., 1988, 1991; Sparks and Ehleringer, 1997). Three possible mechanisms account for the negative influence. The first is associated with changes in leaf morphology with temperature (Körner and Diemer, 1987; Körner et al., 1991). Low temperatures cause plants to increase their leaf thickness (Körner et al., 1988; Hultine and Marshall, 2000), which leads to a longer CO_2_ diffusion distance from the ambient to intercellular air space; as a result, the *c*_i_/*c*_a_ ratios decrease and more ^13^C is enriched in plants (Körner and Diemer, 1987; Zhu et al., 2010); the second is that water viscosity increases with decreasing temperature and this hinders the flux of water in plants. Consequently, plant water potential decreases, resulting in partial stomatal closure and increased δ^13^C (Smith et al., 1984; Cernusak et al., 2013); finally, isotopic kinetic fractionation is expected to increase with decreasing temperature (Guo, 1984). In addition, several mechanisms have been proposed to account for the positive effect of temperature. A decline in temperature usually results in a reduction in enzyme activity and photosynthetic rate (Beerling, 1994), leading to decreased CO_2_ assimilation and consequently a lower growth rate. Under such circumstances the intercellular CO_2_ concentration (*c*_i_) will possibly increase if the ambient CO_2_ concentration (*c*_a_), stomatal conductance (*g*_s_), and mesophyll conductance (*g*_m_) all hold constant; we therefore expect a reduction in δ^13^C with decreasing temperature. In addition, decreasing temperature leads to a lower air-to-leaf water vapor pressure deficit, resulting in an increase in *c*_i_/*c*_a_ ratios and a decrease in δ^13^C values owing to stomatal opening. Lower temperatures reduce dark respiration in plants. Plants enrich ^13^C during dark respiration; thus, lower temperatures result in less ^13^C enriched by plants. ^13^C enrichment in plants with temperature can be also explained by temperature effects on soil moisture through evaporation which is closely associated with soil moisture; relative to precipitation, soil moisture is more directly related to plant δ^13^C. Therefore, I argue that the above all mechanisms are reasonable, and the variations in plant δ^13^C with temperature reflect the combined effects of multiple mechanisms.

Irradiance can also affect carbon isotope discrimination (Δ). Plants exposed to low photon flux densities exhibit higher *c*_i/_*c*_a_ and higher discrimination owing to low photosynthesis rates (Ehleringer et al., 1986). However, all sampling in the present study was carried out at unshaded sites; furthermore, the study area is characterized by abundant sunshine; the data from the Wulumuqi Meteorological Observatory (WLMQ) shows that the sunshine time is 2,645 hyr^-1^ and irradiance is 513.98 Jcm^-2^yr^-1^; irradiance is not the growth-limiting factor. Thus, even if variations in irradiance with elevations have a contribution to the altitudinal trend of plant δ^13^C, the contribution can be neglected.

Air pollutants, such as ozone or sulfur dioxide, have also been suggested to affect carbon isotope discrimination in plants. Pollution may play a role in the *c*_i_/*c*_a_ ratio by inhibition of photosynthesis and/or plants’ adjustment of stomatal conductance. Darrall (1989) argued that the change of *c*_i/_*c*_a_ ratio with pollution level is more complicated, because stomatal closure leads to a decrease in the *c*_i_/*c*_a_ ratio, whereas inhibition of photosynthesis increases the *c*_i_/*c*_a_ ratio. The net result depends on which effect is dominant. Both an increase and decrease in δ^13^C values have been reported in previous observations for the plants exposed to pollutants (Martin and Sutherland, 1990; Elsik et al., 1993; Saurer et al., 1995; Savard et al., 2004; Čada et al., 2016). In this study, all sampling sites were located far from cities and human habitats, with a limited effect of air pollution. Thus, the altitudinal variations in the plant δ^13^C after accounting for the effects of precipitation and temperature (**Figure 5**) mainly reflect the effect of changes in atmospheric pressure with altitude. The results suggest a negative effect of atmospheric pressure on plant δ^13^C.

The effect of atmospheric pressure has been examined in previous studies. Körner et al. (1991), Kelly and Woodward (1995), and Zhu et al. (2010) suggested that decreased atmospheric pressure resulted in an increase in plant δ^13^C, whereas the experimental investigation on *Nardus stricta* by Morecroft and Woodward (1990) showed a positive effect of atmospheric pressure on δ^13^C. Sparks and Ehleringer (1997) argued that a decrease in atmospheric pressure could not account for the altitudinal variation in plant δ^13^C. Relative to other field investigations, the present study may yield a more accurate relationship between atmospheric pressure and plant δ^13^C by accounting for the effects of precipitation and temperature.

Körner et al. (1991) proposed that atmospheric pressure affects foliar carbon isotope discrimination Δ through variations in the pCO_2_ (CO_2_ partial pressure or concentration) and pO_2_ (O_2_ partial pressure or concentration), and that the effect of atmospheric pressure is primarily attributed to pO_2_. Farquhar and Wong (1984) showed that Δ decreased with decreasing pO_2_. Decreased pO_2_ may reduce the oxygen inhibition in photosynthesis but exert no effect on stomatal conductance. Therefore, *c*_i_/*c*_a_ ratios and Δ decrease (Körner et al., 1991). Meanwhile, decreased pO_2_ would also reduce the respiratory rate of plants, and cause a decline in the internal CO_2_ concentration derived from the respiration. As a result, *c*_i_/*c*_a_ ratios and Δ decrease.

The relationship between δ^13^C and pCO_2_ has been studied intensively, and most C_3_ plants showed that Δ increased with increasing pCO_2_ (e.g., Treydte et al., 2009; Wang and Feng, 2012; Schubert and Jahren, 2013, 2015; Cui and Schubert, 2016). McCarroll et al. (2009) proposed two kinds of plant responses to increasing pCO_2_: passive and active responses. The passive response suggests that elevated pCO_2_ does not affect photosynthesis or stomatal conductance; under this case, the increment of *c*_i_ equals that of *c*_a_, resulting in an increase in *c*_i_/*c*_a_ values, thereby increasing Δ. The active response means that photosynthesis and/or stomatal conductance varies with pCO_2_, whereas the *c*_i_/*c*_a_ values remain constant, and therefore, Δ remains unchanged. Wang and Feng (2012) proposed a third response in which *c*_i_ stays constant with rising pCO_2_, resulting in a reduction in both *c*_i_/*c*_a_ ratios and Δ. However, this response is used by only some plants.

## Conclusion

C_3_ plants grown on both sunny and shady slopes of Mount Tianshan characterized by an arid and semiarid climate do not exhibit an altitudinal trend of foliar δ^13^C. The observation confirmed our hypothesis that altitudinal variation in δ^13^C may be negative or unchanged for plants grown in arid and/or semiarid regions. Water availability exerted a significant effect on plant δ^13^C in areas with water stress; resulting in a different altitudinal variation in plant δ^13^C in arid and/or semiarid regions from the general pattern observed in humid areas. After accounting for the effects of temperature and precipitation, the residual δ^13^C exhibited a strong positive correlation with altitude on the both slopes, which suggested that atmospheric pressure had a negative effect on plant δ^13^C.

## Author Contributions

GW designed the study. ZC and GW wrote the paper. GW, YJ, and ZC collected the samples. ZC measured the data.

## Conflict of Interest Statement

The authors declare that the research was conducted in the absence of any commercial or financial relationships that could be construed as a potential conflict of interest.
