# Supplementary material for: Foliar δ13C Showed No Altitudinal Trend in an Arid Region and Atmospheric Pressure Exerted a Negative Effect on Plant δ13C
Source: Front Plant Sci. 2017 Jul 4;8:1070. doi: 10.3389/fpls.2017.01070 (PMC5495824; doi:10.3389/fpls.2017.01070)
Supplement: Supplementary file 1 [file Presentation_1.PDF]

Supplementary Material

Foliar $\delta^{13}C$ showed no altitudinal trend in an arid region and atmospheric pressure exerted a negative effect on plant $\delta^{13}C$

Zixun Chen, Guoan Wang*, Yufu Jia

College of Resources and Environmental Sciences, China Agricultural University, Beijing, China

Correspondence:

Guoan Wang

gawang@cau.edu.cn

Supplementary Figures

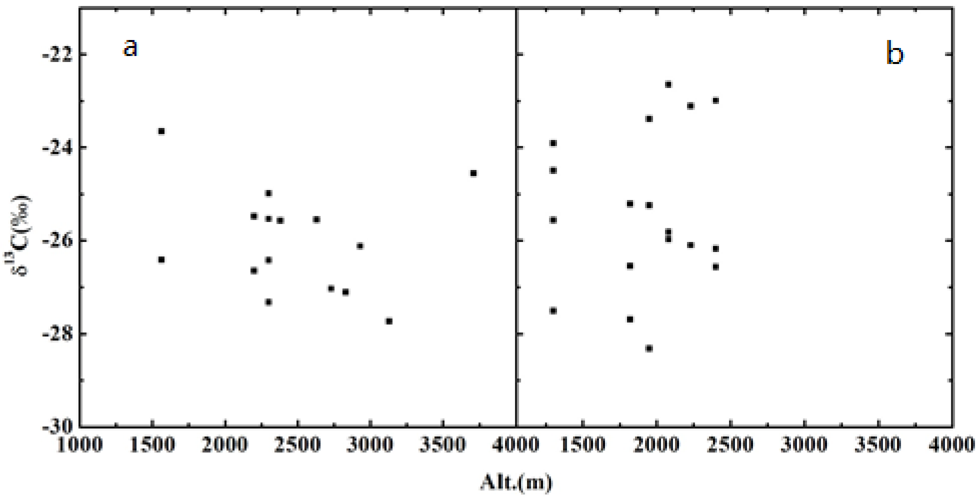

Supplementary Figure 1 Carbon isotope of the woody plants on the shady slope (a) and the sunny slope (b) varies with altitude.

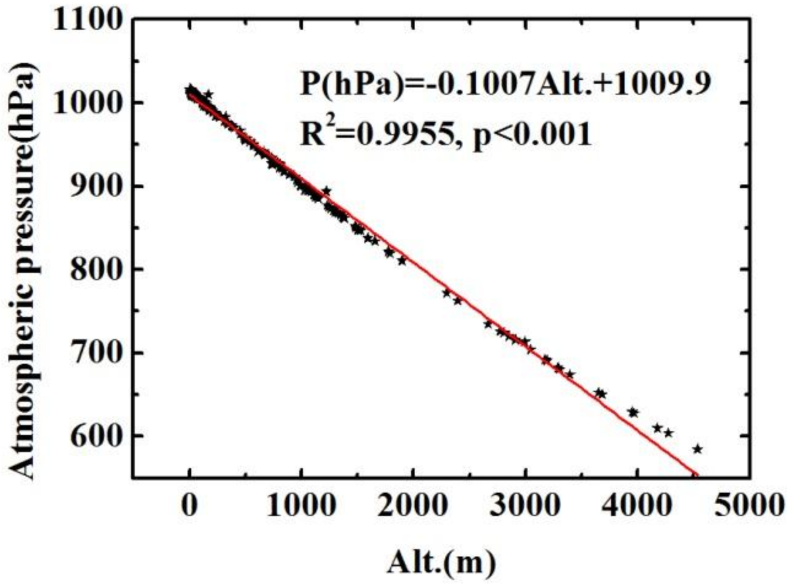

Supplementary Figure 2 The relationship between atmospheric pressure and altitude derived from 193 meterological stations across the Chinese Mainland.
